# Rebuilding Lives: Mental Health and Family Dynamics in Victims of Armed Conflict

**DOI:** 10.1192/j.eurpsy.2026.11671

**Published:** 2026-07-31

**Authors:** D. Sánchez-Pabón, K. Munera-Luque, Z. Miranda Sánchez

**Affiliations:** Magdalena, Universidad Cooperativa de Colombia, Santa Marta, Colombia

## Abstract

**Introduction:**

Armed conflict has been one of the main public health issues for decades in Colombia, exacerbating poverty and social inequality, particularly through demographic changes derived from the forced displacement of many victims. These phenomena have had a direct impact on mental health, well-being, and personal development in social dimensions, especially within the family.

**Objectives:**

To identify the relationship between mental health and family dynamics in victims of armed conflict in Santa Marta

**Methods:**

The research was conducted under a quantitative correlational design, based on a convenience sample of 110 victims of armed conflict in the city of Santa Marta, aged between 18 and 93 years (M = 42.91; SD = 14.86). Various instruments were used, including the characterization form, Family APGAR, and SRQ for the measurement of variables.

**Results:**

Regarding family functioning, 58.2% of families were classified as functional, while 25.5% presented mild dysfunction, 7.3% moderate dysfunction, and 9.1% severe dysfunction. Concerning the presence of psychological symptoms, 46.4% showed symptoms of depression, 51.8% of distress, 29.1% of psychosis, 3.6% epilepsy, 5.5% problematic alcohol use, and 15.5% suicidal ideation. Spearman’s correlations revealed significant associations among several dimensions of psychological distress. Depression was strongly related to distress (rho = .728; p < .001), psychosis (rho = .538; p < .001), and suicidal ideation (rho = .523; p < .001). Distress also showed moderate correlations with psychosis (rho = .498; p < .001) and suicidal ideation (rho = .338; p < .001). Moreover, poorer family functioning was associated with higher levels of suicidal ideation (rho = –.201; p = .035).

**Conclusions:**

A significant relationship was identified between mental health and family dynamics of victims of armed conflict, showing that their lived experiences generate psychological, social, and emotional consequences that affect their overall well-being.

**Disclosure of Interest:**

None Declared

